# The Influence of Negative Emotion on the Simon Effect as Reflected by P300

**DOI:** 10.1155/2013/516906

**Published:** 2013-12-26

**Authors:** Qingguo Ma, Qian Shang

**Affiliations:** ^1^School of Management, Zhejiang University, No. 38 Zheda Road, Hangzhou 310027, China; ^2^Neuromanagement Lab, School of Management, Zhejiang University, No. 38 Zheda Road, Hangzhou 310027, China

## Abstract

The Simon effect refers to the phenomenon that reaction time (RT) is faster when stimulus and response location are congruent than when they are not. This study used the priming-target paradigm to explore the influence of induced negative emotion on the Simon effect with event-related potential techniques (ERPs). The priming stimuli were composed of two kinds of pictures, the negative and neutral pictures, selected from the International Affective Picture System (IAPS). The target stimuli included chessboards of two color types. One was red and black the other one was green and black. Each chessboard was presented on the left or the right of the screen. The participants were asked to press the response keys according to the colors of the chessboards. It was called the congruent condition if the chessboard and the response key were on the same side, otherwise incongruent condition. In this study, the emotion-priming Simon effect was found in terms of RT and P300. Negative emotion compared with neutral emotion significantly enhanced the Simon effect in the cognitive process, reflected by a larger difference of P300 latency between the incongruent and congruent trials. The results suggest that the induced negative emotion influenced the Simon effect at the late stage of the cognitive process, and the P300 latency could be considered as the reference measure. These findings may be beneficial to researches in psychology and industrial engineering in the future.

## 1. Introduction 

The Simon effect, a classical action mechanism, plays an important role in human action control. It may be used to measure the operational performance in manufacturing industries. In everyday life or in industrial operations, efficiency is more likely to be reduced and the severe accidents were more likely to happen for the operators in conditions that involve stimulus-response incompatibility [1, 2]. The classical Simon effect refers to the finding that the reaction time is slower when the stimulus and response locations are incongruent than when they are congruent [3–5]. It is supposed to demonstrate an automatic impact that stimuli sometimes seem to be able to evoke interfering action tendencies against people's will [6].

De Jong et al. [7] constructed a theoretical framework, the dual-route model, to illustrate the classical Simon effect. It states that there are two separate routes with different functions from perception to action. In the intentional (conditional) route, the correct response is purposely selected. While in the automatic (unconditional) route, the response ipsilateral to the target stimulus is automatically activated. When the locations of stimulus and response are congruent, the same response is automatically activated through the unconditional route and selected through the conditional route. Therefore, RT is fast. In contrast, when the locations of stimulus and response are incongruent, RT is slow because the ipsilateral response of stimulus is firstly activated by the unconditional route and then the correct response will be executed through the conditional route. The theory concludes that the Simon effect resulted from the spatial conflict and interference in cognitive process [8, 9].

Emotion has a global effect on the cognitive process [10]. De Houwer and Eelen [11] introduced an affective Simon task in which participants were asked to respond to emotion-laden words (e.g., FRIEND) according to their grammatical status, for example, responding “POSITIVE” to nouns and “NEGATIVE” to adjectives. They found a congruency effect that the reaction time was shorter when word valence matched the response (e.g. responding “POSITIVE” to “FRIEND”). Altarriba and Basnight-Brown [12] found that only negative emotion words produced the congruency effect when the emotion-laden words were replaced with emotion words (e.g., replacing enmity by hatred) in the affective Simon task. However, in these studies, the stimulus and the response are affectively related, rather than spatially. The spatial relevance has been focused on by classical Simon effect [11], while the impact of emotion on the classical spatial Simon effect is still unclear.

When presenting irrelevant emotional and neutral stimuli before a simple cognitive task, researchers usually find slower response in conditions that are preceded by emotional stimuli compared to conditions preceded by neutral stimuli [13, 14]. This emotional interference is attributed to the preferential processing of emotional information [15, 16] and the resources competing when stimuli are in the same sensory modality [17–19]. In particular, people seem to be more sensitive to negative emotions which occupy more cognitive resources [20, 21]. Ma et al. [22] found that the induced negative emotion could elicit larger conflict and mismatch than the induced neutral emotion in the categorizing process. Cohen et al. [15, 23] showed that the conflict monitoring process was significantly influenced by irrelevant negative information. Thus, we supposed that the classical Simon effect which results from the spatial conflict and interference in cognitive process may be influenced by induced negative emotions.

Event-related potential (ERP) with high temporal resolution is an important measure of perceptual and cognitive processes to the stimuli [24]. P300 is a positive ERP component with peak latency around 300 ms after the onset of stimuli [25]. In the Simon task, Melara et al. [9] indicated that the congruency effect was closely associated with P300 component. Incongruent trials evoked smaller P300 amplitudes [9, 26, 27] and delayed P300 latencies [9, 28, 29] in comparison with congruent trials, suggesting that P300 component reflected the resolution of spatial conflict during the process of classifying the event [9, 30]. Among them, according to the context-updating model, P300 latency was a more sensitive measure of conflict resolution since it was closely related to the time of revising one's model during the categorization process of task-relevant events [8].

Based on previous studies, a priming-target paradigm in which the emotional pictures appeared as priming stimuli followed by the target stimuli was used in this study. We hypothesized that the induced negative emotion tended to enhance the spatial Simon effect in the cognitive process, and P300 could be taken as the ERP component reflecting such impact.

## 2. Methods

### 2.1. Subjects

Nineteen right-handed undergraduates or graduates from Zhejiang University were recruited in this experiment. The average age of subjects (11 females) was 21.96 ± 2.40 years. All subjects reported normal or corrected-to-normal visual acuity. No subject had a history of current or past neurological or psychiatric illness. They provided informed consent prior to the study and were paid after the experiment. Our study was approved by the Ethics Committee of Neuromanagement laboratory of Zhejiang University.

### 2.2. Stimuli

The priming stimuli consisted of 40 negative and 40 neutral pictures which were selected from the International Affective Picture System (IAPS) [31] for use in this study. (Neutral pictures were IAPS slides 7000, 7002, 7004, 7006, 7009, 7010, 7020, 7025, 7035, 7041, 7042, 7050, 7052, 7053, 7055, 7056, 7057, 7059, 7080, 7090, 7100, 7130, 7150, 7161, 7175, 7185, 7186, 7187, 7188, 7190, 7192, 7211, 7217, 7224, 7233, 7235, 7236, 7547, 7705, 7950. Negative pictures were slides 1019, 1050, 1200, 1201, 1205, 1275, 1300, 1932, 2375.1, 3005.1, 3015, 3016, 3180, 3181, 3301, 3530, 3550, 6212, 6230, 6313, 6315, 6350, 6370, 6510, 6560, 8485, 9250, 9252, 9254, 9423, 9428, 9429, 9430, 9433, 9440, 9480, 9594, 9635.1, 9810, 9902). These pictures have been showed to be effective to induce negative and neutral emotions (negative stimulus: valence *M* = 2.61, SD = 1.55; neutral stimulus: valence *M* = 5.05, SD = 1.10). Based on the research by Vallesi et al. [32], the target stimuli, 4 × 4 red-and-black or green-and-black chessboards, were presented to the left or right of a central fixation cross on white background, and a 4 × 4 black-and-white chessboard always appeared as a filler opposite to the side of the target stimuli [32].

### 2.3. Procedure

**Figure 1:**
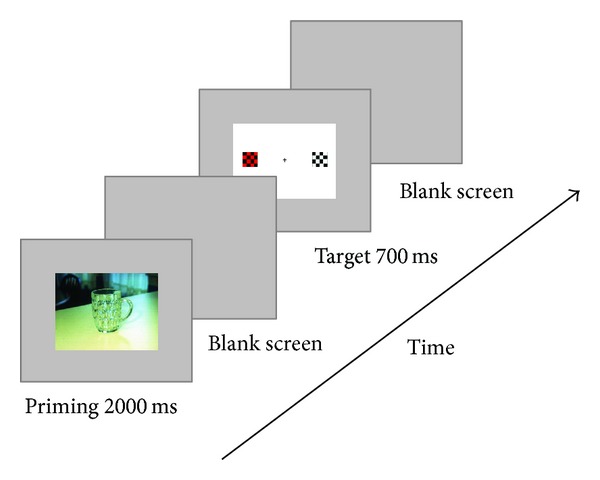
The experimental procedure which illustrates the sequence of stimuli in a trial. Each trial began with a priming stimulus which was an emotional picture selected from the IAPS. Then, the target stimulus which was a red-and-black or green-and-black chessboard, presented to the right or left of a central fixation cross with a black-and-white chessboard always appeared as a filler opposite to the side of the target stimulus. Subjects were required to press the response keys according to the colors of the targets.

This experiment used a priming-target paradigm (see Figure 1). Subjects were informed about the procedure and signed an informed consent form at the beginning of the experiment. During the experiment, subjects were comfortably seated in a sound-attenuated room with a computer keyboard in their hands. The stimuli on the computer screen were 90 cm away from subjects. There were 4 blocks of 80 trials each. Two of the four blocks consisted of negative pictures and the other two were neutral pictures. In each trial, the emotional picture (priming stimulus) was presented with a duration of 2000 ms followed by a random interval ranging from 500 ms to 700 ms. Then the target stimulus appeared for 700 ms with a response deadline of 1000 ms. During the task, the response key on the computer keyboard was the left key (“Z”) to the red-and-black chessboard and the right key (“M”) to the green-and-black [32]. It was called the congruent condition if the chessboard and the response key were on the same side, otherwise incongruent condition. Participants were asked to respond as quickly and accurately as possible. The response hands to press the color-key were counterbalanced across participants. The sequences of emotional priming (negative versus neutral) were presented randomly.

### 2.4. Data Analysis

Electroencephalogram (EEG) was recorded (band pass 0.05–100 Hz, sampling rate 500 Hz) with Neuroscan Synamp2 Amplifier (Scan 4.3.1, Neurosoft Labs, Inc. Sterling, USA), using an elastic cap of 64 Ag/AgCl electrodes mounted according to the extended international 10–20 system. All recordings were referenced to left mastoid. The vertical electrooculograms (EOG) were recorded with one pair of electrodes placed above and below the left eye and the horizontal EOG with another pair of electrodes placed 10 mm from the lateral canthi. EOG artifacts were corrected off-line for all subjects. Electrode impedances were maintained below 5 kΩ throughout the experiment.

Electroencephalogram recordings were extracted from −200 to 800 ms time-locked to the onset of the target stimulus, with −200 to 0 ms as baseline. Electrooculogram artifacts were corrected using the method proposed by Semlitsch et al. [33]. Trials with peak-to-peak deflection exceeding ±80 *μ*V were excluded from averaging. Data were digitally filtered with a low pass filter at 30 Hz (24 dB/Octave). To validate the hypotheses of this study, the within-subjects repeated measure ANOVAs were used to the behavioral and ERP data.

## 3. Results

### 3.1. Behavioral Data

The acceptance rate (AR) and reaction time (RT) were analyzed separately using 2 (congruence: congruent versus incongruent) × 2 (emotion: negative versus neutral) repeated measure ANOVA. The analysis for AR only revealed a significant main effect for congruence (*F*(1, 18) = 9.199, *P* < 0.01), but neither a main effect of emotion (*F*(1,18) = 0.52, *P* > 0.05) nor an interaction between congruence and emotion (*F*(1,18) = 0.000, *P* > 0.05) was found. The AR was smaller in the incongruent condition (*M* = 98.8%, SE = 0.003) than in the congruent condition (*M* = 97%, SE = 0.006).

The ANOVA on RT produced a main effect of congruence (*F*(1,18) = 63.36, *P* < 0.001). The RT was longer in the incongruent trials (*M* = 469.73 ms, SE = 14.10) than in the congruent trials (*M* = 434.17 ms, SE = 12.77). The main effect of Emotion (*F*(1,18) = 0.22, *P* > 0.05) and the interaction between congruence and emotion (*F*(1,18) = 0.003, *P* > 0.05) were not significant.

### 3.2. ERP Data

**Figure 2:**
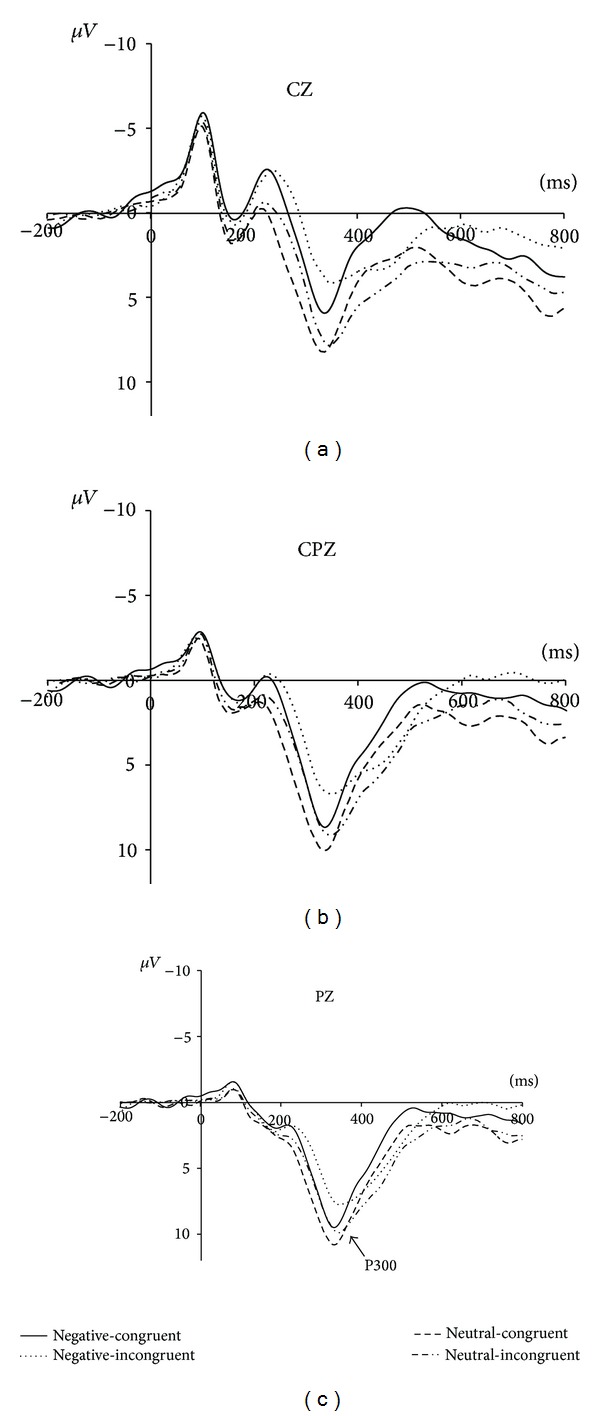
ERP results. Grand averages of P300 in response to the four categories of stimuli (negative-congruent, negative-incongruent, neutral-congruent, and neutral-incongruent).

The grand average waveforms of P300 were shown in Figure 2. According to previous studies on Simon effect [27, 28], P300 was distributed on the parietal/central brain regions. Therefore, the following 9 electrode sites (C3, CZ, C4, CP3, CPZ, CP4, P3, PZ, and P4) were selected for statistical analysis. The time window (300–360 ms) of P300 latencies and amplitudes calculated in this study was consistent with previous studies on Simon effect [27, 34].

The ANOVAs of 2 (congruence: congruent versus incongruent) × 2 (emotion: negative versus neutral) × 9 (electrode) for latency and amplitude of P300 were performed. For the latency, a significant main effect was found for congruence (*F*(1,18) = 22.99, *P* < 0.001), whereas no significant main effect was observed for emotion (*F*(1,18) = 2.48, *P* > 0.05) and electrode (*F*(8,18) = 2.173, *P* > 0.05). The latency was longer for the incongruent trials (*M* = 356.80 ms, SE = 7.14) than for the congruent trials (*M* = 333.74 ms, SE = 4.52). There was also a significant interaction between Congruence and Emotion (*F*(1,18) = 9.90, *P* < 0.01). Simple effects analyses revealed that the difference of P300 latency between the congruent and incongruent trials was larger in the negative priming condition (*F*(1,18) = 25.74, *P* < 0.001) than in the neutral priming condition (*F*(1,18) = 8.19, *P* < 0.05). All the other interactions were not significant.

The ANOVA for the P300 amplitude showed significant main effect for congruence (*F*(1,18) = 15.94, *P* < 0.005) and emotion (*F*(1,18) = 16.11, *P* < 0.005), respectively. The amplitude of P300 for the congruent trials (*M* = 8.33 *μ*V, SE = 0.97) was significantly larger than for the incongruent trials (*M* = 6.95 *μ*V, SE = 1.04). The induced negative emotion elicited significantly smaller P300 amplitude (*M* = 6.60 *μ*V, SE = 0.99) than the neutral emotion (*M* = 8.68 *μ*V, SE = 1.06). The main effect of electrode and all the interactions were not significant.

## 4. Discussion

The classical Simon effect emphasizes the interference of irrelevant feature (i.e., spatial location) on response selection [3–5]. What influences this interfering effect is an important issue. With ERP measurement, we examined the emotion-priming Simon task to explore the neural evidence of emotional impact on the spatial Simon effect. Our results showed that the Simon effect was significant in terms of RT and P300, while the incongruent condition had longer RT, smaller P300 amplitude, and longer P300 latency. Moreover, we found the neural evidence of emotional impact on the Simon effect that the negative emotion compared to the neutral emotion elicited larger difference of P300 latency between incongruent and congruent trials.

The classical Simon effect showed that the RT in the incongruent trials was longer than in the congruent trials [5, 32]. In the current emotion-priming Simon task, the Simon effect was also significantly observed. This finding indicates that the emotion-priming Simon effect involved the same behavioral feature as the classical Simon effect. According to the dual-process model of Simon effect [7, 32], when the side of target stimulus and response hand was incongruent, the ipsilateral response of target firstly activated by the unconditional route and then the correct response would be selected through the conditional route, resulting in a slow RT. In comparison, when the target stimulus and response hand appeared on the same side, the conditional and unconditional routes were activated consistently, resulting in a fast RT.

Our ERP results of emotion-priming Simon effect reported that P300 was modulated by congruence between stimulus and response locations; that is, both smaller amplitude and longer latency of P300 were observed in the incongruent condition as compared to the congruent condition, consistent with the studies on the classical Simon effect [9, 26–29]. It seems that the emotion-priming Simon effect was associated with the same electrophysiological signature as was the classical Simon effect, suggesting that emotion has no impact on the cognitive processes.

However, this initial conclusion seems to be misleading. Since our more important finding was that the negative emotion significantly enlarged the difference of P300 latency between the incongruent and congruent trials, demonstrating that the induced negative emotion influenced the cognitive process of Simon effect in terms of P300 latency. Previous studies demonstrated that induced negative emotion could elicit larger conflict than did the induced neutral emotion in the stimulus classifying process [4, 5, 31]. According to the context-updating model [8], P300 latency was a sensitive measure which reflected the revising time of one's model during the process of conflict resolution. In the present study, when the induced emotion was negative, the spatial conflict in the incongruent trials tended to be enlarged that the P300 latency was longer. Thus, negative emotion compared to neutral emotion showed larger difference of P300 latency between the incongruent and congruent trials. The finding indicates that the induced negative emotion influenced the Simon effect at the late stage of the cognitive process, and the P300 latency could be considered as the reference measure.

In conclusion, the current study used the priming-target paradigm to explore the emotional influence on the spatial Simon effect. The Simon effect was clearly observed in the measurements of RT and P300. The induced negative emotion significantly influenced the cognitive process of Simon effect in terms of P300 latency; that is, negative emotion compared with neutral emotion evoked larger difference of P300 latency between the incongruent and congruent trials. It suggests that the P300 latency could be considered as a reference measure to assess the emotional influence on the spatial Simon effect. These findings may be beneficial to studies in psychology and human action control in industrial engineering in the future.
